# The role of the enrolling clinician in emergency research conducted under an exception from informed consent

**DOI:** 10.1007/s11017-025-09710-9

**Published:** 2025-04-01

**Authors:** Katherine Sahan, Ethan Cowan, Mark Sheehan

**Affiliations:** 1 The Ethox Centre, Oxford Population Health, University of Oxford, Big Data Institute, Old Road Campus, Roosevelt Drive, Oxford, UK; 2 Icahn School of Medicine at Mount Sinai, New York, USA

**Keywords:** Research ethics, Therapeutic emergency research, EFIC, Ethical enrolment, Informed consent

## Abstract

The Exception from Informed Consent (EFIC) permits patient enrolment into therapeutic emergency research where obtaining informed consent is challenging. Yet this fails to resolve a core ethical conflict in the research and has generated controversy. This is because existing justification and practice has relied on applying EFIC *per study*—a wholesale permission to enroll irrespective of circumstance—instead of *per patient*. Our novel justification for enrolment centers on applying EFIC per patient, which empowers the enrolling clinician to judge whether to enroll patients with an Exception. This contrasts with the idea that clinician judgment is surplus to the judgements already made by institutions in deciding the research may proceed. Instead, we show that enrolling clinician’s judgment is ethically significant and should not be overlooked: attending to this strengthens the research ethically and reduces controversy. There should be a bigger role for the clinician in the research enrolment space.

## The core ethical conflict in emergency medicine research

Conducting emergency research with the prospect of direct therapeutic benefit presents a core conflict in research ethics between respecting participant autonomy and improving emergency care. On one side of the conflict, the setting of the research is urgent, the potential therapeutic window for intervention is narrow, and its participants are suffering from life-threatening conditions. This makes the possibility of prospective informed consent to the research either extremely challenging or impossible and so could undermine respect for their autonomy^1^. On the other side, conducting research in this context and on human participants is vital to improve the evidence base for future emergency care. Reflecting this conflict, US regulators banned certain types of emergency research from 1993 to 6 [1] but in 1996 developed a special mechanism of “exception from informed consent” or “EFIC” to regulate and permit emergency research without consent [2–4]. The EFIC mechanism in emergency research constitutes a major challenge to conventions in research ethics which typically require informed consent to research with the prospect of therapeutic benefit which is greater than minimal risk. Mechanisms similar to EFIC are now the standard in many countries’ regulations and international guidelines [5–7].

The EFIC mechanism in emergency research is a regulatory recognition and accommodation of the ethical conflict which the research presents. It recognises that in the majority of cases, individual patient consent will not be possible and exceptions from informed consent (EFICs) will occur. However, it must accommodate the unlikely eventuality that consent, proxy consent, or obtaining relevant objections from family members may be possible despite the emergency context. It does this by making the use of EFICs conditional on certain other clauses which require investigators to check that informed consent (from patients or proxies) is not feasible. In the small minority of cases where consent is feasible it should be obtained, so respecting patient autonomy as far as context and circumstances allow [8]. The mechanism is accompanied by certain “additional protections” given the (assumed majority) absence of consent [9]. These include (in the case of the US): (a) public disclosure campaigns; (b) community consultation measures, and (c) attempts to consent the patient or proxy for “continued participation” after the emergency EFIC enrollment stage [10].

In what follows, we explore the idea that despite having mechanisms such as EFIC, the core ethical conflict involved in conducting this kind of research persists. First, we describe a persistent ethical controversy surrounding the conduct of and enrollment to emergency research approved under EFIC regulations, using two contrasting examples to illustrate how this arises in practice. This leads to our paper’s central claim about the importance of the clinician’s real-time, per patient engagement with the ethical conflict presented at enrolment. Second, we contrast this claim with existing bioethics strategies for justifying the research, showing how they miss the importance of the clinician’s judgment. Finally, we argue for a novel justificatory strategy, making clinician judgment a central feature in applying Exceptions to individual patients.

## Persistent controversy

Since the Federal Drug Administration’s (FDA’s) approval of EFIC regulations in 1996 there has been persistent ethical controversy over its use. This controversy is evident in numerous quarters both academic and public.

In academic quarters, there has been considerable debate about the appropriateness and ethical justification for EFIC-approved emergency research. Writing in 2002, Andrew McRae and Charles Weijer’s account of a moral justification for emergency research characterizes emergency patients as “arguably the most vulnerable class of potential research participants” because they are unable to give informed consent [10, p. 1146]. They suggest stringent protections are needed in addition to existing EFIC regulations [10]. Eight years later in Largent et al.’s ethical justification of emergency research using a consent waiver, the authors note that “[w]hether emergency research without initial consent should be permitted remains controversial” and that “no ethical justification for conducting emergency research without informed consent has been widely accepted” [11, p. 669]. More recently in 2018, two reviews of US EFIC-approved emergency research studies note further aspects of controversy. Firstly a review from Klein et al. examines how the EFIC Regulations were interpreted and applied to a number of published emergency research studies, finding that investigators have frequently struggled to explain (at least in published accounts) why their study merited an approval using an EFIC mechanism [12]. They conclude that “[s]ince research without consent is an ethically sensitive issue and not widely understood, better justification of its needs within the presentation of the research itself may educate the general medical community and also reduce concerns about whether or not the regulations are being properly applied” [12, p. 1176].

A second 2018 review from Feldman et al. conveys how this controversy is evident in public as well as academic quarters. It notes that while there are supporters of emergency research approved under EFIC, “the efficacy of [EFIC] regulations and the actual risk/benefit balance in EFIC trials remain a source of controversy” [13, p. 1606], citing a 2017 US House Committee investigation into whether surrogate decision makers are being appropriately involved in the research. They go on to describe three areas of further concern which affect “respect for persons” and “public trust” in the research. Firstly, questionable low withdrawal rates. On Feldman et al.’s view, it is surprising that withdrawal rates should be so low (less than 1%) in the EFIC studies they reviewed. This is despite possible explanations for low withdrawal rates in the form of (a) high mortality rates, (b) completion of primary interventions before patients or surrogates had opportunity to withdraw, and (c) the general drive from regulation to include all primary endpoint data in the analysis, even when patients withdraw after the primary endpoint, to reduce bias. They also report that investigators presented withdrawal options in a variety of ways, some more accessible to patients than others. This causes Feldman et al. to call for standardization in withdrawal processes “in a way that balances the need to minimize scientific bias and maximize respect for patients and their autonomy” [13, p. 1611].^2^ A second area, concerning for respect and trust reasons, is described as racial disparity in recruitment, referring to an overrepresentation of African Americans in EFIC trials, possibly due to correlation of academic medical centers in urban areas with large African American populations. Thirdly, the authors cite a lack of transparency about reporting of consent data such as data on how many patients or surrogates refuse consent, or how many are enrolled using consent or proxy consent.

The persistent academic and public controversy surrounding EFIC might suggest there is something fundamentally unethical in conducting EFIC-approved emergency research. While we do not think the research is unethical, we acknowledge that conducting it ethically will always remain a challenge due to the underlying core ethical conflict (i.e., respecting participant autonomy *versus* improving emergency care), and how this emerges in practice. Where we do see room for improvement is in how to think about and manage the conflict in practice within EFIC-approved studies, which in itself might help lessen instances of controversy in the research. Not addressing controversy risks eroding public trust and causing significant harm to the field of emergency research approved under EFIC Regulations and investigators’ own career prospects. The work of this paper is therefore to argue for how practically to engage with the conflict in everyday EFIC-approved research enrolment situations in a way that mitigates against controversy.

### Example 1: HEAT-PPCI (not engaging with the conflict)

Here we show how controversy arises when clinicians do not engage the core ethical conflict (i.e., respecting participant autonomy *versus* improving emergency care). The example is the 2014 UK HEAT-PPCI trial, a 1:1 open label randomized controlled trial (RCT) of two antithrombotic drugs, bivalirudin and heparin, in acute myocardial infarction (heart attack). The trial sought to test the “relative safety and efficacy” [15, p. 1850] of the two antithrombotics, both in routine use, by a direct comparison. The trial received the UK equivalent of an EFIC approval from the IRB since the majority of participants would have been unable to consent prospectively (a) due to their medical condition (acute myocardial infarction associated with ST segment elevation or STEMI) and (b) because of their emergency presentation.^3^ In their subsequent trial publication, investigators reporting using the EFIC mechanism (which they referred to as “a policy of delayed consent”) for the following reason:

Very sick, elderly, or frail patients, those with low socioeconomic status, women, and individuals from ethnic minorities are often not approached for study participation. We obtained ethical approval for a policy of delayed consent and set ourselves the demanding goal to randomise all eligible patients entering the catheterisation laboratory in the setting of a PPCI activation—“Every Patient, Every Time” [15, p. 1850].

This shows that EFIC approval was given in order to manage consent arrangements ethically, but investigators also saw it as an opportunity to improve the quality of the science by reducing enrolment biases and so increase the generalizability of results. But to use their EFIC approval in this way, investigators would have needed to interpret it liberally—treating it as a wholesale permission to enroll each and every eligible patient without consent.

This approach to enrolment and the ethics of the trial were heavily criticised at the 2014 American College of Cardiology conference, with one expert panellist commenting that:

There is general consensus on the panel that we hope we’ve not entered an era where we are experimenting on humans without their consent again [18].

And another that:

I would hold the leaders of your organization accountable to make sure that the public and the community knows what’s going on. There is an absolute social contract that we make to our patients to make sure they are not subject to research without their permission...I’m extremely bothered by the fact that you entered patients into the research study without getting their position [sic] [18].

So, while investigators had a clear sense of how the trial was ethical under EFIC, the panel suggested two major ethical flaws: (1) that investigators had instrumentalized patients; and (2) that this might erode public trust.

Within the panel discussion and on other occasions, investigators justified their treatment of patients as ethical given (1) the closeness of the trial to usual care since the drugs randomized were approved and in routine use, and (2) the greater societal benefit arising from increased generalisability of results (via liberal use of EFIC at enrolment) [18, 19]. However, neither the panel nor investigators seem aware of how the EFIC mechanism should be practically implemented to mitigate against concerns of instrumentalizing patients. Namely, although permission to use the EFIC mechanism is granted *per study*, Exceptions are only legitimately used *per patient*, after the enrolling clinician makes several judgements about feasibility of obtaining consent or of soliciting objections [3, 20]. This mitigates against instrumentalizing patients as merely a means to the trial’s ends by treating each patient as a person worthy of respect despite her likely inability to provide consent. This is consistent with the Respect for Persons principle from Belmont, a close relative of respect for autonomy which affords ethical protections in the case of non-autonomous individuals [21, 22].

## Our central claim: use EFIC per patient not per study

This leads to the central claim of our paper: that enrolling clinicians (or other enrollers^4^) whose studies are EFIC-approved should operationalize the approval by using Exceptions *per patient* not *per study* (as happened in HEAT). By judging whether to apply an Exception per patient, clinicians properly engage with the core ethical conflict of respecting autonomy versus improving care through research. They do this by respecting the patient as a person worthy of moral consideration, even though it is not possible to respect her autonomy by obtaining her consent. This makes Respect for Persons a key principle underpinning our claim [21, 22].

To be clear, when we refer to the making of judgements about applying an Exception, we do not mean judging a patient’s clinical eligibility for enrolment by checking against a pre-defined set of inclusion or exclusion criteria from the study protocol. Rather, the judgements we are interested in are made after a patient is judged clinically eligible to take part. These judgements are informed by considerations generated by the individual enrollment encounter between patient and clinician, considerations which are related to appropriateness of enrolment. In non-emergency settings, such ethical considerations would be generated by a particular patient’s or proxy’s ideas about what matters to him/her in respect of deciding about enrollment to the research. The relationship between patient and clinician (and family members) would further inform and mediate these considerations and any enrollment judgements following from them. In the emergency setting of an EFIC study, those considerations do not disappear, but many are inaccessible due to difficulties presented by context. This presents a dilemma about how one should act to honor this enrollment encounter and aspects of the patient-clinician relationship, making ethical enrollment judgements and retaining a measure of respect for patients. The kind of judgement we are describing in our central claim is thus about preserving these ethical enrollment considerations as far as possible, respecting the person even if not his/her autonomy.

Using a fictional example, we now develop this central claim further to say that through engaging with the core ethical conflict, clinicians will sometimes encounter patients with reference to whom there are reasons not to deploy an Exception. The implications of this are that it may be appropriate to exclude them from a study, even though the study is EFIC-approved, and the patients may meet clinical eligibility criteria.

### Example 2: Ken (engaging with the conflict)

Here we imagine an enrolling clinician, Ken, working in an EFIC-approved trial. Ken judges that an Exception should not be applied in the case of two patients, X and Y (Table 1).

**Table 1:** Fictional example of engaging with the conflict in an EFIC-approved trial

Ken is conducting a study of a novel therapeutic for traumatic head injury. The trial compares the new therapy against a widely adopted but unsatisfactory existing standard of care. The broader scientific community does not have the evidence to be sure that the new treatment is better and this trial is designed to help settle the question and so disturb clinical equipoise. Head injury patients eligible for the trial have high levels of morbidity and mortality but the risks the trial adds (over and above the context) are minimal. The trial is EFIC-approved. Its inclusion and exclusion criteria are clearly specified with an age range of 18–80 years old.	
Two eligible patients are brought to the ER having suffered serious head trauma and are bleeding into their skulls. X is a female and aged 75. Y is a 19-year-old male with physical signs of a developmental disorder. Both fulfil clinical eligibility criteria as given in the trial protocol. There is no accessible information about their wishes (e.g. DNR order or advance directive), nor are family or friends immediately accessible. Both patients must immediately either be enrolled in the trial or excluded and treated with standard of care. The trial is approved by EFIC and so Ken can technically enroll both patients but has concerns. In the case of X, his clinical sensibilities make him uneasy about including her in this way in the research as she looks frail and he is not sure how she will cope with the new therapy. In the case of Y, he is unsure about putting someone so young, and with so much at stake, in an experimental protocol, without a proper chance to talk it through with him, his parents, or someone who knows him well. Ken thinks he might be hesitating because he is comfortable with the standard of care and knows it well,even though he cannot know for certain whether it is the better option.	

In the above example, it is clear that the time-pressured context and the study-wide EFIC approval could invite automatic enrolment of patients like X and Y. Yet the context also gives rise to reasons which are generated from Ken’s standpoint as he encounters and observes these particular patients. Such reasons justify pause for thought about whether to include them in the trial. Some reasons may well track the types of enrolment biases given in the HEAT example above (e.g., co-morbidity, frailty, gender). This indicates that while enrolment bias may be a problem for the science, there may nevertheless be a social reality to why some groups may not be approached for, or agree to research, and in certain cases this may give reason not to enroll certain patients. Other reasons may be connected with Ken’s role as a clinician: a caring intuition towards a frailer candidate (X), a sense that the threshold for finding and consulting with proxies should be higher in some cases (Y) [20], or just enough of a hesitation about running the experimental protocol in X and Y’s case when the standard of care seems the “safer,” or at least better known option [23]. To be clear, the reasons behind the judgements here are fine-grained considerations about the appropriateness of putting a given patient through the experimental protocol. The judgements are neither driven by a reactionary, gut instinct towards enrolment, nor are they connected to presence or absence of personal equipoise, or an undermining of clinical equipoise (though enrolment may not be right for X or Y, it may yet be right for the next patient, Z).

Example 2 develops our central claim because it shows that respect for persons within EFIC-approved studies depends on individually considering their suitability for enrolment. X and Y, though they may be unconscious or unable to communicate verbally with Ken are nevertheless ‘speaking’ to Ken (his intuition, his sensibility, his professional experience) in a way that discourages enrolment. These considerations justify Ken standing aside from any supposed policy (such as HEAT’s “Every Patient Every Time” policy) or institutional pressure to recruit all patients. An incidental advantage of excluding X and Y (with good reasons) is that Ken proves the patients are not used merely as a means to scientific ends (the instrumentalization point).

Overall, proper engagement with the ethical conflict, as demonstrated by Ken, might help to reassure patients and public groups that their interests are being better protected in emergency research approved under EFIC regulations. This might have the indirect benefit of fostering public confidence and trust in the research and lessening potential controversy, provided that any resulting exclusions happen justifiably and not for so-called tokenism.

## Strategies which contrast with our central claim

Our central claim stands in contrast with two existing strategies from the bioethics literature, which argue that making general assessments about emergency patient cohorts is sufficient for enrolling them justifiably.

Firstly, a strategy from McRae and Weijer who address the special vulnerability of emergency patients through a comprehensive, separate analysis of therapeutic and non-therapeutic risks which is designed to protect them from excessive risk exposures in research [10, 24]. The risk analysis follows a strict procedure and is performed pre study approval by the clinical community (subjecting therapeutic risks to the test of clinical equipoise) and the IRB (deciding whether non-therapeutic risks have been minimised). This strategy does not—at least not explicitly—require any particular assessment of individual patients from an enroller like Ken.

A second strategy comes from Emily Largent et al. who justify enrolling patients to emergency research using the Reasonable Person Standard or RPS [11]. In emergency care, the RPS is used to judge that an incompetent emergency patient would agree to being treated (similar to implied consent for emergency care). This Standard presupposes that in emergency situations, the diverse interests of most individuals would converge towards interests for welfare and survival, suggesting most would choose to be cared for [11, p. 670]. This Standard is then applied to justify enrollment to emergency research using five “conditions,” many relating to predefined risk–benefit parameters, which ensure equivalence between research and care. Of these conditions, only the third (Condition 3: no conflicting preferences) seems focused on assessing the individual patient. As with the strategy in McRae and Weijer, Largent et al.’s strategy is to make generalized judgements pre study approval, both about risk–benefit, and about what a typical emergency patient’s reasons and welfare considerations would be. This contrasts with actively judging each patient in context, as Ken does.

The strategies here reiterate the wrong-headedness of over-relying on the ‘use EFIC per study’ approach. There is a concerning tendency to front-load all the ethical assessments pre study approval, leaving little to be decided at the ‘per patient’ level. This is perhaps to mitigate an imagined difficulty in making judgments in the urgent setting. But such over-reliance is misguided on several fronts. Firstly, it ignores clauses within the US EFIC Regulations which require investigators to (a) make per patient checks of whether proxy consent is feasible, (b) prepare informed consent documents and procedures for use where patient/proxy consent is feasible and (c) commit to attempting to contact family members to assess potential objections to a patient’s enrolment [8, 20].^5^ Secondly, it makes the institutional force of the EFIC approval oppressive, both to individual patients and to researchers like Ken. By this we mean that enrollers like Ken might feel discouraged from expressing their own per patient enrollment judgements if the ‘use EFIC per study approach’ is over-emphasized in combination with the time-pressured context. If his judgements are so discouraged and suppressed, patients are made potentially worse off and treated with less respect than they should be. Thirdly, over-relying on this approach is misguided because it under-values Ken’s intuitions and skills in making reasonable enrollment judgements despite the urgent setting, since if we assume Ken is a good emergency clinician, he is also well accustomed to taking reasonable decisions quickly amidst uncertainty.

We turn instead to a third type of strategy which starts to resemble our central claim, by taking the context of individual patients into account.

### A strategy that resembles the central claim: examine the enrolment context for objections to enrolment

As described above, Largent et al.’s strategy is mainly to rely on generalized judgements to make enrolment decisions. However, their third condition, ‘No conflicting preferences,’ indicates some focus on an individual patient assessment: under this condition one should check that enrolment will not conflict with particular patients’ preferences. However, even within this individual check, the checking process retains a generalized thrust since what counts as a conflicting preference is decided in advance (e.g., having a DNR, wearing an opt-out bracelet). While this will capture a small range of pre-defined reasons for exclusion, it is not responsive to the many other reasons which may arise in context. Thus, although Largent et al.’s account acknowledges the importance of context, it does not take active steps to respond to context or actively interrogate it. A related paper by Vorholt and Dickert is more promising in this respect [20]. Like Largent et al., the paper examines conflicts at the level of individuals, but does so through an active interrogation of context, focusing on when to solicit and honor objections to enrollment. These objections may come from conscious patients, family members, or a patient’s roommate [20]. The account goes on to argue that “uninformed refusals”—cases where patients object on less than sufficient understanding of what enrollment means, or where proxies object based on partial knowledge of patient preferences – should yet have ethical force and support the exclusion of such patients.

This paper—plus the Largent et al. check of no conflicting preferences—amounts to a more promising strategy in respect of our central claim: clinician judgments matter in enrolment decisions and depend on assessing individual persons and context. Nevertheless, within Vorholt and Dickert it is unclear whose judgments—clinician or proxy—are more ethically significant to justify enrolment. In our view, there are judgments of a first (clinician) and second (clinician plus objecting proxies or friends) order at work in Vorholt and Dickert. The first-order judgment is about when clinicians should solicit objections, i.e. whether to bring objections ‘into play’ in the enrolment context or whether the setting is too urgent and clinicians need to make enrolment decisions about the patient on their own. The second-order judgment is about when clinicians should honor proxy objections already solicited or given. To us, the Vorholt & Dickert account focuses only on this second-order judgment and in doing so undersells the ethical significance of the first-order judgment. This ethical significance is driven by the urgent context of enrollment, because of which it is likely that a) patients will be unconscious, or unable to engage in any way about the prospect of research; and b) proxies will be unavailable. This will mean that often the clinician will be solely responsible for enrollment or exclusion questions (as in the example of Ken above). Additionally, even where proxy opinions or objections are in the mix (moving towards a second-order judgement), it is still the clinician’s first-order judgement which determines whether to bring objections into play. This elevates the importance of first-order judgments over second-order ones—the clinician is still ultimately responsible for enrollment decisions in most cases.

## Our novel strategy: empower enrolling clinicians to use EFIC per patient

Overall, none of the existing strategies above sufficiently recognize or value clinicians’ judgment in deciding whether using an EFIC is appropriate on a per patient basis. In contrast, we have argued that clinician judgment has a separate, unrecognised importance within questions of emergency enrollment or participation, even where there is approval to enroll under EFIC. This gives rise to a novel strategy which makes investigator judgment central to the justifiability of enrollment decisions: in addition to existing institutional decision-making processes (e.g., IRB approvals, equipoise judgments), investigators should feel empowered to use their judgment to decide whether an Exception applies per patient. Importantly, although feeling so empowered may be taken to track what standardly happens in clinical research practice—investigators can and do exclude even eligible patients from studies—to our knowledge there has been no explicit articulation of this and *why it is justified* in the EFIC case.

Our strategy is based not only on the insights from Example 2 (Ken) above, but also on related discourses within the literature. Firstly, the account by Vorholt and Dickert evokes our strategy by noting that while “EFIC creates a default of enrolment, it does not create an obligation to enrol an individual” [20, p. 20]. Secondly, a paper by Miller and Weijer—not specific to emergency research—argues that “REC approval does not entail the moral or legal acceptability of enrolling particular patient-subjects in research” [25 p. 545]. They make this observation as part of analyzing the differences between institutional obligations to society in research versus researchers’ obligations to individual patients. Miller and Weijer argue that the principle of clinician’s judgment should play a key role within the research enrollment context. This clinical judgment principle is broadly articulated as specifying a “duty of care that is, in turn, firmly rooted in the moral and legal theory of trust relationships” and as protecting “agent-relative welfare interests” of patients [25, p. 546]. Upholding this principle matters because it balances out caring and research obligations to the patient. The principle obtains despite prior decisions at the level of institutions (such as equipoise judgments, or IRB approval) which might indicate that because the research is permissible, so is wholesale permission to enroll. Thirdly, Garland et al. have recently argued that any duty of clinicians to participate in pragmatic clinical trials is defeasible for multiple reasons relating to clinicians’ judgment about including certain patients in certain trials [26]. This can be because of inwardly personal considerations (conscience), professional limits (lack of skill or time), or relational factors (trust or rapport with patients) which might justify a clinician not including her patients [26].

We have previously agreed with Garland et al. that the duty is defeasible and also suggested that acceptable reasons for not “participating” or enrolling patients in PCTs might split into (1) patient-oriented and (2) professionally-oriented considerations [23]. In the emergency research case, the patient-oriented consideration describes what may happen when a clinician encounters a patient at enrolment. Even with pre-approval to enroll the patient, the clinician should re-confirm to him/herself that this patient should be enrolled. Alternatively, they should also feel empowered to exclude as part of this relational encounter, perhaps feeling that the particular patient would not suit the experimental protocol. The professionally-oriented consideration refers to a more practical set of reasons based on resources, professional competence, and capabilities. For example, clinicians might object to trial participation when including some patients in a trial might affect their ability to care for other patients; being excessively tired, time-pressured, or lacking clinical confidence in a different or novel technique may be acceptable reasons for not participating.

### Objections to our strategy

One reasonable objection to our strategy is that what we are proposing is unlikely to change enrollment practices or make a difference to enrolment rates. On the point about changing practices, we accept that our novel strategy amounts to supporting the expression of subtle, internal kinds of ethical judgements and development of moral “craft” [27]. This could be a complex kind of practice or practice change to convey but should not detract from the worth of what we are proposing. We also anticipate our strategy will meet a practice need and will resonate with emergency research practitioners who have experience of feeling conflicted between the ‘per study’ and ‘per patient’ force of the EFIC mechanism and who have felt disempowered to use their own judgement.

On the point about making a difference to enrollment rates, as we noted with Feldman et al.’s concern about questionable low withdrawal rates in EFIC studies (see the second section above and footnote 2), our concerns and our arguments are less about the ends or *outcomes* of EFIC enrollment. Rather, we focus on whether *processes* and treatment of patients at enrollment are ethical and justifiable. Therefore, adopting our proposed strategy does not mean necessarily being able to see differences in which patients are enrolled or excluded, but we would expect a difference in how processes are conducted and decisions taken pertaining to enrollment or exclusion. Such process differences would not perhaps be readily observable but could be elucidated with behavioural or attitudinal research about investigator decision-making. Better processes can strengthen procedural legitimacy (for example by improving accountability for enrollment judgements), so giving rise to improved public confidence and trust in emergency research and lessening controversy [28].

A second objection to our strategy is that giving a greater role to clinician judgment could be a threat to the scientific validity pursued by examples such as HEAT above. This is also the starting position of Garland et al. who maintain that the duty of clinicians to participate in PCTs should be the ‘default.’ In answer to the first objection, we think scientific validity is important and that clinicians should think carefully and give good reasons for excluding patients, particularly from groups who are often excluded from trial participation. However, we think the clinician’s judgment is still required to make an enrollment or exclusion decision. This is because the judgment process respects a patient as a person and specifies a duty of care owed to this patient in an enrolment situation. It is additionally (but incidentally) because a clinician who undertakes her duties in this way is in a good position to stem any controversy which could arise, because her actions promote public trust in the treatment of research participants.

A third objection to our strategy might focus on the quality of reasons given for excluding patients, claiming for example, unacceptable reasons (called, for example, reasons of “conscientious objection” [26]) might be smuggled in under the guise of our strategy. For example, a clinician with a political objection to research, to his/her institution, or to mechanisms like clinical equipoise, might try to exclude patients via our strategy. Equally, a clinician’s judgment might not be ideologically based, but in a paraphrase of Friedman, be “unduly sensitive to the vagaries of intuition and opinion” [25. p. 546]. In answer to this second objection, we do not think the clinician needs to have a political agenda to experience Ken’s above concerns, but neither can we dismiss those concerns as vague, reactionary, or whimsical. Rather it is the context and his duty as a clinician and researcher which motivates him and gives rise to robust reasons. This motivation and reasoning are independent of Ken’s views about research, equipoise, and the like. Indeed, Ken could have easily applied an Exception to five previous patients but still hesitated over X and Y. Such hesitation is more than pure gut instinct or whimsy (as Friedman might suggest)—there is significance and ethical force to it—but we grant that specification will not be simple and again will depend on several subtle contextual factors.

While the specification question is difficult, we can envisage some general situations and considerations which might relate meaningfully to making decisions about non-enrollment. Firstly, the situation where the patient may not *do well*, despite fulfilling inclusion criteria. This brings the clinician’s judgement and sense of that particular patient’s well-being within an experimental protocol to bear on the decision-making. Secondly, the patient whose situation or demeanor *gives off a general sense* that they or their caregivers would object to their enrollment. In this situation, patient or caregiver values and choices seem not to be aligned with enrollment. Thirdly, a situation where a *lack of clinician bandwidth* to manage this patient, perhaps in the way that they might require, as well as other patients on that shift, is relevant to the enrollment decision. This situation speaks to their personal ability to cope with an already intense clinical practice and research context.

In each of the situations above, we have used colloquial expressions in italics to convey the subtle, situational forces that might press upon clinician judgement as part of a kind of ‘inner ethical monologue’ about enrolment. It is forces like these and how they contribute to reasonable clinician judgement which make them ethically significant and valuable.

## Conclusion

We have presented and defended a novel justificatory strategy for enrolling patients to emergency medical research approved under an Exception from Informed Consent (EFIC). Firstly, we detailed a core ethical conflict in this kind of research, that of respecting patient autonomy versus improving emergency care. We then noted that US regulators and others have launched EFIC mechanisms which allow participants to be enrolled without consent, but that these accommodate the core conflict, rather than resolve it. Secondly, we described a persistent controversy when conducting EFIC-approved studies, which risks harming the field of emergency research or investigators’ own career prospects. Using the example of the HEAT PPCI trial, we argued that this controversy relates to a lack of practical engagement with the core ethical conflict in the research. This led to our central claim in the second section: that enrolling clinicians should use EFIC *per patient* not *per study* (i.e. not treating an EFIC approval as wholesale permission to enroll every participant irrespective of individual circumstance). This is because using EFIC per study fails to treat patients as individuals worthy of respect and so risks instrumentalizing them for study ends. We developed our claim with the example of Ken who engages with the core conflict and decides (reasonably) not to enroll two patients. On our view, Ken’s judgements are respectful of patients as persons and are promoting of public trust, so mitigating the potential for controversy.

Thirdly, we contrasted our central claim with existing strategies which rely too heavily on generalized judgements to justify patient enrolment to EFIC-approved studies. As well as generalized decision-making (e.g., equipoise judgements in the clinical community and IRB-determined judgments about patient risk or reasonableness), one should also value the skills and individual judgment of clinicians to make appropriate enrolment decisions about individual patients, despite the challenges of the emergency setting (and noting that emergency clinicians are accustomed to taking reasonable decisions quickly amidst much uncertainty). Fourthly, building on literature which distinguishes institutional level and clinician level duties towards individual participants, we described our novel strategy for justified enrolment in EFIC-approved studies. This strategy justifies the empowerment of clinicians to use their judgement at the level of individual patients about when and whether to apply Exceptions, after which we dealt with a number of objections to the strategy.

In sum, we do feel there is a bigger decision-making role for the clinician in the emergency research enrollment space. When properly empowered and equipped to reason about whether to grant Exceptions in individual patients, enrolling clinicians have great potential to uphold nuanced, context-sensitive ethical practice, and reduce instances of controversy arising from EFIC-approved research studies.

## Footnotes

1 We describe consent as either challenging or impossible to reflect clauses within the US EFIC Regulations requiring investigators to check whether consent is impossible in every case. In the very few cases where consent is possible, they are required to obtain it. See also footnote five.

2 While we do not think low withdrawal *rates* are necessarily problematic, we agree with Feldman et al. that getting withdrawal *procedures* right is ethically important in EFIC research for reasons similar to those we go on to give in our account of ethical enrollment below. Namely, the emergency context and provision of EFIC Regulations to accommodate that context should nevertheless aim to uphold as far as possible standards for procedures designed to support ethical participation. This includes procedures for enrolment and withdrawal. This is to avoid concerns about respect for patients and trust in the research. For example, see this interesting discussion about the UK Paramedic 2 trial which argues that not informing surrogates of their relatives’ inclusion in emergency trials (for their information generally or, we assume, to allow for withdrawal options) can risk losses of public trust [14]).

3 The 2nd Amendment (2006) of the UK Clinical Trials Regulations 2004 would have been the relevant instruments underpinning this approval. The wording of the legislation is as follows: “(6) Sub-paragraph (7) applies if treatment is being, or is about to be, provided for a. subject who is an incapacitated adult as a matter of urgency and, having regard to the nature of the clinical trial and of the particular circumstances of the case—(a) it is also necessary to take action for the purposes of the clinical trial as a matter of urgency; but (b) it is not reasonably practicable to meet the conditions [for consent or proxy consent] set out in paragraphs 1 to 5 of Part 5. (7) Where this sub-paragraph applies, paragraphs 1 to 5 of Part 5 [consent or proxy consent arrangements] shall not apply in relation to the subject if the action specified in sub-paragraph (6) is carried out in accordance with a procedure approved by an ethics committee or by an appeal panel appointed under Schedule 4 [where an unfavourable original ethics committee opinion was appealed] at the time it gave its favourable opinion”’ [16, 17].

4 We expect clinicians (physicians, nurses) to mainly enroll to research of this kind, but other responsible healthcare professionals (e.g. paramedics) could also be enrollers

5 The relevant clauses from the US EFIC Regulations are as follows (our headers and emphases added in bold) [8]: **Check whether proxy consent is feasible.** “The proposed investigational plan defines the length of the potential therapeutic window based on scientific evidence, and the investigator has committed to attempting to contact a legally authorized representative for each subject within that window of time **and, if feasible, to asking the legally authorized representative contacted for consent within that window rather than proceeding without consent.” **(21 CFR 50.24(a)(5)). **Check whether consent is feasible.** “The IRB has reviewed and approved informed consent procedures and an informed consent document consistent with § 50.25**. These procedures and the informed consent document are to be used with subjects or their legally authorized representatives in situations where use of such procedures and documents is feasible.”** (21 CFR 50.24(a)(6)). **Check for objections to patient enrolment.** “If obtaining informed consent is not feasible and a legally authorized representative is not reasonably available, **the investigator has committed, if feasible, to attempting to contact within the therapeutic window the subject's family member who is not a legally authorized representative, and asking whether he or she objects to the subject's participation in the clinical investigation.** The investigator will summarize efforts made to contact family members and make this information available to the IRB at the time of continuing review” (21 CFR 50.24(a)(7)(v)).
